# The effects of lumbar stabilization exercises with and without jaw movements in non-specific low back pain (A randomized controlled trial)

**DOI:** 10.12669/pjms.40.6.9208

**Published:** 2024-07

**Authors:** Muhammad Khan, Hamayun Zafar, Syed Amir Gilani, Waqas Ahmed Farooqui, Ashfaq Ahmad

**Affiliations:** 1 Muhammad Khan, PT, MSPT Institute of Physical Therapy & Rehabilitation, Jinnah Sindh Medical University, Karachi, Pakistan. University Institute of Physiotherapy, The University of Lahore, Lahore, Pakistan; 2 Hamayun Zafar, PT, PhD Dept. of Rehabilitation Sciences, College of Applied Medical Sciences & Medical Research Chair, King Saud University Riyadh, Saudi Arabia. University Institute of Physiotherapy, The University of Lahore, Lahore, Pakistan; 3 Syed Amir Gilani, MBBS, DMRD, MPH, PhD(Ultrasound), PhD (Public Health), University Institute of Physiotherapy, The University of Lahore, Lahore, Pakistan; 4 Waqas Ahmed Farooqui, MSc, PhD School of Public Health, Dow University of Health Sciences, Karachi, Pakistan; 5 Ashfaq Ahmad, University Institute of Physiotherapy, The University of Lahore, Lahore, Pakistan

**Keywords:** Exercise Therapy, jaw movement, low Back Pain, lumbar Stability, temporomandibular Joint

## Abstract

**Objective:**

This study aimed to investigate the added effect of jaw clenching on the efficacy of lumbar stabilization exercises to manage chronic non-specific low back pain.

**Methods:**

This randomized controlled trial was conducted at the Sindh Institute of Physical Medicine and Rehabilitation (SIPM&R) Karachi from April 2021 to April 2023. Eighty patients with chronic non-specific low back pain participated in this study. Forty patients each were randomly allocated to the lumbar stability exercise (LSE) group’ and the lumbar stability exercise with teeth clenching (LSETC) group. Patients in both groups performed respective exercises twice weekly for 12 weeks. The Numeric Pain Rating Scale (NPRS), Roland Morris Disability Questionnaire (RMDQ), and Pressure Biofeedback Unit (PBU) were used to assess pain, disability, and muscle endurance respectively. Data were collected at the baseline, after six weeks and 12 weeks of intervention. A p-value of <0.05 was considered statistically significant.

**Results:**

Both groups showed statistically significant improvements in pain, disability, and muscle endurance. Upon further stratification, participants aged 20-30 years in the LSETC group showed significantly higher scores than the LSE group for NPRS, RMDQ, and PBU after 12 weeks. Overall, the LSETC group showed relatively higher improvement in mean scores for NPRS, RMDQ, and PBU than the LSE group.

**Conclusion:**

Lumbar stabilization exercises with and without jaw movement are effective for the treatment of chronic non-specific low back pain. The addition of teeth clenching enhanced the effectiveness of lumbar stability exercises, especially in young adults.

***Trial Registration:*** Clinicaltrials.gov (NCT04801212), Prospectively registered on March 16, 2021.

## INTRODUCTION

Low back pain (LBP) is one of the complex bio-psychological health conditions that challenge clinicians in the developed world.^1^ The cost of this condition is quite high and data from European countries have suggested the cost varies between 0.1-2%.^2^ However; no data is available from low and middle-income countries. The indirect costs such as Physician visits, disability benefits, and loss of work productivity contribute to over 80% of the total costs in social welfare states.^3^ A study conducted in Karachi Pakistan has reported that 58% of healthcare providers experienced low back pain in which 27.4% had moderate to severe disability.^4^ Low back pain and temporomandibular joint dysfunction (TMJD) may occur simultaneously which may cause great limitations in functional activities. To reduce the chances of the progression of the condition to more chronic stages it might be necessary to pay attention to the treatment of temporomandibular joint dysfunction (TMJD) and low back pain together.^5^

Several studies have shown the positive effects of jaw movement on maintaining an upright standing posture and enhancing vertical jump activities in sports.^6^ In a recent study teeth clenching showed antagonist muscle activation simultaneously to increase strength, kinetic, and exercise performance during functional spinal assessment.^7^ Most of such studies were carried out on healthy subjects or athletes and it remains to be seen whether the addition of jaw movement would enhance motor control in patients with somatic pain conditions. Among these chronic low back pain (CLBP) is the most common condition with a debilitating nature. Weakness in trunk muscles, poor coordination, and motor control contribute to the development of low back pain.^8^ Lumbar stabilization exercises are effective in the management of chronic nonspecific low back pain as compared to other conventional therapies.^9^

A recent systematic review in the area also supports the findings of the previous studies.^10^ Based on the pieces of evidence from the association between jaw movement and trunk muscles 6 it could be assumed that the additional effect of jaw movement could be beneficial for the correction of altered motor control and reduce pain in patients with nonspecific chronic low back pain. Therefore, it will be interesting to investigate whether performing lumbar stability exercises along with teeth clenching will enhance lumbar stability muscle activation through concurrent activation.^6^ To the best of our knowledge, no previous study has evaluated the effects of teeth clenching along with lumbar stability exercise intervention for non-specific low back pain. Thus, this study aimed to investigate the effect of teeth clenching along with lumbar stability exercises for the treatment of non-specific chronic low back pain.

## METHODS

**Fig.1:**
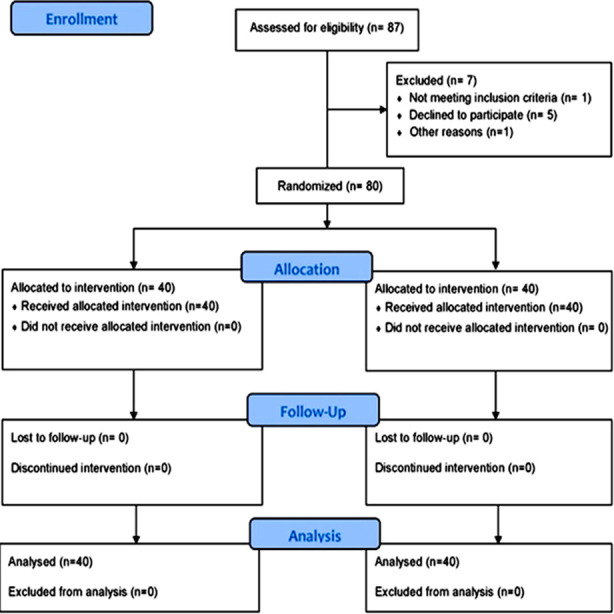
Flow chart of the recruitment, randomization and follow up of participant.

This randomized controlled trial was conducted at the Sindh Institute of Physical Medicine and Rehabilitation (SIPM&R) Karachi from April 2021 to April 2023. The trial is registered on www.ClinicalTrial.gov with registration identification: NCT04801212. A total of 80 participants (40 males and 40 females) with chronic nonspecific low back pain who met the inclusion criteria were recruited using a non-probability purposive sampling technique for the study (Fig.1).

### Ethical approval

It was obtained from the institutional review board (IRB) University of Lahore with reference No: IRB-UOL-FAHS/373/XI/2021.

The inclusion criteria were both male and female participants aged 20-45 years who had chronic low back pain (for more than 12 weeks) with or without leg pain. The exclusion criteria were: known temporomandibular joint pathology, a serious spinal pathology (infective or inflammatory diseases of the spine, tumors, cauda equine syndrome, fractures, disc disease, stenosis, spondylolisthesis, lumbar spondylosis, and compressed nerve root), a history of previous spinal surgery, or presence of any co-morbid health conditions such as heart and lung pathology that may affect active participation in the exercise program. The sample size of at least 13 subjects per group was calculated using PASS version 15 (NCSS, Kaysville, Utah, USA) software, based on the independent samples t-test’, allowing unequal variance with 99% confidence interval, 99% power of the test, the mean of RMDQ score in the intervention group (1.7 ± 2.4) and the control group (7.9 ± 3.3).^11^ To increase the power of the study, we included 40 participants in each group including a 15% drop-out.

Those participants who met the inclusion criteria were given a brief outline of the study. The participants willing to participate in the study were invited for baseline assessment and were given more information about the study process. After the baseline assessment and written informed consent, the participants were randomly and equally allocated to two groups by using computer-generated random numbers in a Microsoft Excel Sheet. The lumbar stabilization exercise (LSE) group performed lumbar stabilization exercises alone consisting of abdominal drawing in supine, alternate self-resisted hip flexion in supine, contracting the abdominal muscles and dropping the alternate leg to the side in supine, contracting the abdominal muscle in sitting and contract the abdominal muscles in standing for initial three weeks. From week four to six the exercises progressed to abdominal drawing in supine, bridging, bridging with alternate straight leg raise, alternate arm raise in the crawling position, and opposite arm and leg raise in the crawling position. Each exercise was carried out for 10 repetitions, three sets, and held for five seconds. The lumbar stabilization exercises with teeth clenching (LSETC) group’ performed lumbar stabilization exercises the same as the LSE group with teeth clenching. The participants were instructed to perform maximal voluntary isometric contraction of the jaw-closing muscles and hold for 10 seconds before performing each exercise in the exercise protocol.^12^ This exercise protocol was adapted from the previous works on static and dynamic stability.^13^,^14^ A total of 12 sessions were performed for six weeks with a frequency of two sessions each week and the duration of each session was 45 minutes. Both intervention groups received electrical heating pads on the back for 15 minutes and the interventions were applied under the supervision of an experienced musculoskeletal physiotherapist. Both groups were instructed to perform the same exercises at home which they carried out during the six weeks of intervention and keep weekly diaries of their home exercises till the final assessment on week 12.

Baseline and post-intervention pain and disability scores were assessed with the Numeric Pain Rating Scale 15 (NPRS), and Roland Morris Questionnaire 16 (RMDQ). Abdominal muscle endurance was measured with a Pressure Biofeedback Unit 17 (PBU). All subjects in each group were re-assessed for pain, disability, and muscle endurance by using the same scales following completion of the 6-week intervention and 12-week follow-up. Pre and post-intervention assessment was carried out by the same assessor who was blind to the treatment allocation. Data were analyzed on IBM SPSS version 26 (IBM Inc., Armonk, NY, USA). The normality of the data was tested for each variable. The association between gender and groups was assessed using a chi-square test. For outcome measures repeated measure two-way ANOVA was applied to evaluate the mean difference within the groups after considering the normality of the data distribution. For pair-wise comparison least significant difference (LSD) test was applied. A P-value of 0.05 or less was considered significant.

## RESULTS

A total of 80 participants completed the study and there was no drop out. Both groups had an equal number of participants. At baseline, there was no significant difference between the two groups in demographic characteristics, (Table-I).

**Table-I:** Demographic characteristics of the participants.

Characteristics	LSE N = 40	LSETC N = 40	P-value
***Gender***			
Male	20 (50.0)	20 (50.0)	>0.99^C^
Female	20 (50.0)	20 (50.0)	
Age (years), mean ± SD	32.2 ± 7.5	31.1 ± 7.3	0.520^I^
BMI (kg/m^2^) , mean ± SD	22.97 ± 3.6	22.95 ± 2.8	0.978^I^

LSE = Lumbar stability exercise, LSETC = Lumbar stability exercise with teeth clenching, ^c^ Chi-square test,

I Independent sample t-test, SD: standard deviation.

### Post-intervention within group outcome measures changes

Both treatment groups showed statistically significant improvements in pain, disability, and muscle endurance (p < 0.05) for all tested outcome measures. However, mean improvements in post-intervention NPRS score, RMDQ score, and PBU were better in the LSETC group as compared to the LSE group, Table-II.

**Table-II:** Post-intervention within-group mean differences in outcome variables.

Outcome Measures	LSE (N = 40)	Δ	Imp (%)	P-value^¥^	LSETC (N = 40)	Δ	Imp (%)	P-value^¥^
***NPRS Score***								
Baseline	5.60 ± 1.6	-	-	-	6.03 ± 1.3	-	-	-
At week 6	3.38 ± 1.4	2.225	39.7	<0.001	3.53 ± 1.5	2.50	41.5	<0.001
At week 12	3.35 ± 1.3	2.250	40.2	<0.001	3.25 ± 1.3	2.775	46.1	<0.001
***RMDQ Score***								
Baseline	9.28 ± 2.7	-	-	-	10.55 ± 3.7	-	-	-
At week 6	6.55 ± 2.5	2.725	29.4	<0.001	7.40 ± 3.4	3.150	29.9	<0.001
At week 12	6.08 ± 2.5	3.20	34.5	<0.001	6.78 ± 3.4	3.775	35.8	<0.001
***PBU (mm/Hg)***								
Baseline	63.78 ± 2.5	-	-	-	63.63 ± 2.8	-	-	-
At week 6	62.25 ± 3.0	1.525	2.4	<0.001	61.68 ± 3.3	1.950	3.2	<0.001
At week 12	61.75 ± 3.8	2.025	3.2	<0.001	61.60 ± 3.5	2.025	3.2	<0.001

¥ P-value on comparison using repeated measures two-way ANOVA with post-hoc (LSD) test, Imp: Improvement, NPRS: Numeric Pain Rating Scale, RMDQ: Roland Morris Disability Questionnaire, PBU: Pressure Biofeedback Unit, LSE: Lumbar stability exercise, LSETC: Lumbar stability exercise with teeth clenching, Δ: mean difference.

### Between the group outcome measures changes

Table III shows the pre-post-intervention between-group differences in the NPRS score, RMDQ score, and PBU values at week six and week 12 follow-ups. No significant between-group differences were found in NPRS score, RMDQ score, and PBU values at the 6^th^ or 12^th^-week follow-up (p>0.05).

**Table-III:** Pre and post-intervention between-group mean differences in outcome measures.

LSE vs LSETC	Baseline		At week 6		At week 12	

	Δ	P-value	Δ	P-value	Δ	P-value
NPRS Score	0.425	0.195	0.150	0.640	0.10	0.738
RMDQ Score	1.275	0.080	0.850	0.207	0.70	0.291
PBU (mm/Hg)	0.150	0.801	0.575	0.424	0.150	0.853

NPRS: Numeric Pain Rating Scale, RMDQ: Roland Morris Disability Questionnaire, PBU: Pressure Biofeedback Unit, Δ: mean difference, LSE = Lumbar stability exercise, LSETC = Lumbar stability exercise with teeth clenching, ^¥^P-value on comparison using repeated measures two-way ANOVA with post-hoc (LSD) test.

### Baseline mean change and improvement (%) of outcome among groups by age

Statistically significant differences were observed in both groups when stratified according to age. Participants aged 20-30 years in the LSETC group showed significantly higher scores for NPRS, RMDQ, and PBU than in the LSE group after twelve weeks. However, at week six, significant differences between the two groups were only observed for RMDQ and PBU, Table-IV.

**Table-IV:** Baseline mean change and improvement (%) of outcome among groups by age.

Age group	20 – 30 years (N = 43)				>30 years (N = 37)			

Intervention	At six weeks		At twelve weeks		At six weeks		At twelve weeks	

Outcome Measures	Imp (%)	Δ	Imp (%)	Δ	Imp (%)	Δ	Imp (%)	Δ
***NPRS Score***								
LSE	38.9	2.158	38.1	2.105	42.3	2.286	42.3	2.381
LSETC	45.6	2.542	50.3	2.792	38.3	2.438	41.6	2.750
Δ (p-value)	0.384 (0.267)		0.686 (0.045)		0.152 (0.626)		0.369 (0.385)	
***RMDQ Score***								
LSE	26.4	2.316	30.3	2.684	33.9	3.095	39.8	3.667
LSETC	33.0	3.0	40.3	3.708	31.7	3.375	35.2	3.875
Δ (p-value)	0.684 (0.019)		1.024 (0.045)		0.280 (0.508)		0.208 (0.710)	
***PBU (mm/Hg)***								
LSE	1.8	1.158	2.0	1.263	2.9	1.857	4.4	2.714
LSETC	3.2	2.0	3.2	2.042	3.0	1.875	3.2	2.0
Δ (p-value)	0.842 (0.005)		0.779 (0.013)		0.018 (0.970)		0.714 (0.355)	

NPRS: Numeric Pain Rating Scale, RMDQ: Roland Morris Disability Questionnaire, Δ= mean difference from the baseline, PBU: Pressure Biofeedback Unit, LSE = Lumbar stability exercise, LSETC = Lumbar stability exercise with teeth clenching, Imp: Improvement.

## DISCUSSION

This study explored the interaction of mandibular muscles and the core muscles of the back. To the best of our knowledge, this is the first study to utilize the addition of jaw movement in conjunction with lumbar stabilization exercises in patients with non-specific chronic low back pain. The results of the present study have suggested that lumbar stabilization exercise with jaw movements leads to comparatively better outcomes than the exercises performed in isolation, especially in individuals aged 20-30 years where significant differences were observed among the LSETC group. Previous studies have also reported a reduction in low back pain after administering treatments targeting the temporomandibular joint.^18^,^19^

In the present study, both groups showed significant improvements in pain, disability, and abdominal muscle endurance at weeks six and 12 (P <0.001). This is in agreement with Chan et al.^20^ study showed that a six-week duration of lumbar stability exercise is an effective intervention in chronic non-specific low back pain. This study followed the participants for 12 weeks and found 6% higher improvements in the LSETC group on the pain scale. The effect of teeth clenching on pain may be due to an inhibitory effect of teeth clenching on the reverberating circuits in the spinal cord which underlie the continued chronic pain. 21 Teeth clenching could activate the deepest core muscles of the lumbar spine such as the multifidus, which take the load off the more superficial muscles like the erector spinae.^21^ An increase in the cross-sectional area of multifidus has also been found to decrease chronic low back pain and the associated disability employing real-time ultrasonic imaging.^22^ Teeth clenching has been known to facilitate the activation of the trapezius, paravertebral, and rectus abdominis muscles.^12^ The observed effects of teeth clenching during lumbar stability exercises to reduce pain and disability and to increase endurance are in line with the concept of concurrent activation potentiation.^23^

In the present study, the mean differences evident between the two groups hold statistical as well as clinical significance. More interestingly participants aged 20-30 years reported significantly greater improvements in all three outcomes in the LSETC group at week 12. Whereas, the situation was reversed in the LSE group in which better outcomes were reported in the participants with ages above 30 years. This phenomenon was observed on both 6-week and 12-week benchmarks, however, the statistically significant differences were only observed in the LSETC group. The reason for this disparity while not ascertained can be attributed to age-related changes which become more evident in the thirties and beyond.^24^

Considering the previously established values of minimal clinically important difference (MCID), the mean difference in NPRS scores in both groups yielded clinically significant differences at both six and 12 weeks.^25^ For RMDQ however, despite showing a statistically significant difference at week six the scores became clinically significant only at 12 weeks, considering the recommended threshold of at least 30% from the baseline.^26^ For the pressure biofeedback unit, a reduction in pressure of at least 4 mm Hg - 5.82 mm Hg has been reported to represent a normal abdominal muscle.^27^

In the present study, however, a maximum mean difference of 2mm Hg was observed in PBU scores from the baseline at 12 weeks. Further studies with the inclusion of outcome measures such as ultrasound imaging will be required to clarify this further. Further explanation of better results in the teeth clenching group could be interconnection between teeth clenching and core muscle activation possibly has its roots in the proximity between trigeminal sensory-motor nuclei and the highly excitable pontine reticular nuclei, which are concerned with mastication and antigravity muscle activation.^28^,^29^ Since these two sets of nuclei lie adjacent to each other, it is plausible to argue that the pontine reticular nuclei receive at least some neuronal input from the neighboring trigeminal nuclei, and vice versa. Such interconnections have already been found between the pontine reticular nucleus and the interneurons surrounding the trigeminal nucleus in the reticular formation.^30^ Teeth clenching is also known to enhance spinal cord anterior horn cell excitability (H reflex) and serves as reinforcement for deep tendon reflexes.^31^

### Limitations

This study focused on measuring the endurance of abdominal muscles in general with PBU. Electromyography (EMG) and Ultrasound scans are also reliable methods for measuring trunk muscle activities which were not used due to the lack of availability of these tools in physical therapy settings.

## CONCLUSION

There are no additional effects of jaw clenching on the efficacy of lumbar stability exercises to manage chronic non-specific low back pain. However, jaw clenching showed some potential to enhance the effectiveness of lumbar stabilization exercises in young adults.

### Recommendations

Future research may include outcome measures such as EMG and ultrasound scans to capture isolated muscle activity during teeth clenching in trunk flexors and extensors muscles.

### Authors Contribution:

**MK:** Topic selection, designed, data collection, & manuscript writing.

**HZ:** Topic selection, designed, manuscript review, supervision.

**SAG:** Editing & review of manuscript, supervision.

**WAF:** Sample size calculation and data analysis.

**AA:** Review and approval of final manuscript.

**MK:** Takes the responsibility and is accountable for all aspects of the work in ensuring that questions related to the accuracy and integrity of any part of the work are appropriately investigated and resolved.
